# Kikuchi-Fujimoto Disease in Pregnancy: A Rare Case With Atypical Presentation and Potential Viral Trigger

**DOI:** 10.7759/cureus.115466

**Published:** 2026-08-30

**Authors:** Nicole Nudelman, Ann K Valencia, Alex Fernandez, Gustavo A Hernandez, Jeffrey Valencia Uribe, Brian G Nudelman

**Affiliations:** 1 Osteopathic Medicine, Kansas City University, Kansas City, USA; 2 Allopathic Medicine, Florida International University, Herbert Wertheim College of Medicine, Doral, USA; 3 Allopathic Medicine, Florida International University, Herbert Wertheim College of Medicine, Miami, USA; 4 Critical Care Medicine, University of South Florida, Pembroke Pines, USA; 5 Cardiology, Memorial Healthcare, Pembroke Pines, USA

**Keywords:** kfd with diffuse lymphadenopathy, kikuchi, kikuchi disease, kikuchi-fujimoto's disease (kfd), s: kikuchi-fujimoto disease

## Abstract

Kikuchi-Fujimoto disease (KFD), or histiocytic necrotizing lymphadenitis, is a rare, self-limiting condition characterized by cervical lymphadenopathy, fever, and night sweats. Although KFD is most commonly reported in young Asian women, cases have emerged globally, including among non-Asian populations. The etiology remains unclear, with viral and autoimmune triggers, such as Epstein-Barr Virus (EBV) and systemic lupus erythematosus (SLE), being commonly proposed. Diagnosing KFD is challenging due to its overlapping symptoms with more severe conditions like tuberculosis, malignancies, and autoimmune disorders. Diagnosis typically requires an excisional lymph node biopsy due to the insufficiency of noninvasive methods.

This case report describes a 29-year-old pregnant African-American female who presented with diffuse cervical lymphadenopathy, B symptoms, and a positive purified protein derivative (PPD) test. Her clinical course included a comprehensive infectious and autoimmune workup, revealing positive EBV IgG, ultimately leading to a diagnosis of KFD via lymph node biopsy. Management focused on supportive care, and she had a favorable outcome with an uncomplicated delivery.

The case underscores the importance of considering KFD in the differential diagnosis of lymphadenopathy, particularly in high-risk populations. It also highlights the need for careful balancing of symptomatic treatment with fetal safety. While KFD generally resolves spontaneously, ongoing monitoring is critical due to its potential association with SLE. This case adds to the limited literature on KFD in pregnancy, emphasizing the importance of early recognition, appropriate biopsy, and multidisciplinary management. Further research is warranted to explore the pathogenesis of KFD, especially its potential link with viral infections like EBV.

## Introduction

Kikuchi-Fujimoto disease (KFD), also known as histiocytic necrotizing lymphadenitis, is a rare but relatively benign condition characterized by cervical lymphadenopathy, night sweats, and fever. Classically, this disorder has been associated with Japanese and other Asian young women; however, isolated cases have been reported in the United States and Europe [1]. While the etiology and pathogenesis of KFD are not well understood, the leading theories regarding the cause of the disease include viral or autoimmune illness [1]. Most notably, an association with systemic lupus erythematosus (SLE) has been described [1].

The pathophysiology of KFD remains unclear, though leading theories suggest that infection, injury, or an underlying autoimmune process triggers T-cell activation, leading to apoptosis and a cytokine storm [1]. This severe inflammatory response is thought to potentially precede malignancy or the development of SLE in affected patients [1].

Noninvasive diagnostic tools, including laboratory tests and imaging, have proven insufficient for diagnosing KFD. Instead, diagnosis relies on excisional lymph node biopsy of an affected node; fine needle aspiration is typically inadequate due to insufficient sample size [1].

Our case describes a rare presentation of KFD in a young pregnant African American female. We review the existing literature to outline the considerations required when managing KFD in pregnant patients and to explore possible explanations for our patient’s presentation.

## Case presentation

We present the case of a 29-year-old African American woman presented to the emergency department with worsening bilateral cervical lymphadenopathy and new-onset pleuritic chest pain. She had initially presented to the emergency department 1 month earlier with cervical lymph node swelling, sore throat, and fatigue. At that time, she reported multiple sick contacts at home, including her two children, who had experienced similar symptoms. She was discharged with amoxicillin-clavulanate and prednisone.

Her medical history was significant for anemia and a previous episode of herpes simplex virus infection. She did not have any prior history of surgeries. She was not taking any chronic medications, and she had no known drug allergies. Family history was noncontributory. She denied tobacco, alcohol, and recreational drug use. Her vaccination history was up to date. She denied recent travel outside the United States, occupational hazard exposure or contact with animals.

During outpatient follow-up after her initial emergency department visit, she had tested positive for both purified protein derivative (PPD) and pregnancy. She subsequently returned to the emergency department because of progressive cervical lymphadenopathy accompanied by pleuritic chest pain and night sweats. She denied fever, weight loss, cough, dyspnea, rash, arthralgias, gastrointestinal symptoms, or any other symptoms.

On presentation, the patient was well appearing, alert, in no acute distress. Her temperature was 36.3 °C, blood pressure 113/69 mmHg, heart rate 77 beats/min, respiratory rate 16 breaths/min, and oxygen saturation 99% on room air. Examination of the head and neck demonstrated multiple tender, enlarged lymph nodes along the posterior cervical chains bilaterally, with associated tenderness of the cervical musculature. Oropharyngeal examination was unremarkable. Cardiovascular and pulmonary examinations demonstrated normal findings. The abdominal examination was normal with no hepatosplenomegaly. Examination of the extremities demonstrated no edema/calf tenderness, and the skin examination revealed no rash or other pertinent findings.

Initial laboratory evaluation demonstrated leukopenia with neutropenia, microcytic anemia, and hypokalemia. The quantitative β-human chorionic gonadotropin level was 24,539 mIU/mL, consistent with pregnancy. D-dimer was elevated at 1.07 mg/L. Urinalysis demonstrated trace leukocyte esterase, ketonuria, microscopic hematuria and pyuria, and squamous and hyaline casts without bacteriuria. Blood and urine cultures subsequently showed no growth. Complete laboratory findings are summarized in Table 1.

**Table 1:** Initial Lab Values ANC: absolute neutrophil count; β-hCG: beta-human chorionic gonadotropin; CMV: cytomegalovirus; CRP" C-reactive protein; EBV: Epstein-Barr virus; ESR: erythrocyte sedimentation rate; fL: femtoliter; g/dL: grams per deciliter; hpf: high-power field; IgG: immunoglobulin G; IgM: immunoglobulin M; LDH: lactate dehydrogenase; MCV: mean corpuscular volume; mg/L: milligrams per liter; mIU/mL: milli-international units per milliliter; mm/h: millimeters per hour; mmol/L: millimoles per liter; PCR: polymerase chain reaction; PPD: purified protein derivative; RBC: red blood cell; U/L: units per liter; WBC: white blood cell; µL: microliter.

Test	Result	Units	Reference range
White blood cell count (WBC)	2.8	× 10³/µL	4.0–11.0
Bands	6.0	%	0–5
Absolute neutrophil count (ANC)	1.17	× 10³/µL	1.5–8.0
Hemoglobin	10.5	g/dL	11.6–15.0 (adult female)
Mean corpuscular volume (MCV)	67.5	fL	80–100
Sodium	135	mmol/L	135–145
Potassium	2.9	mmol/L	3.5–5.2
Quantitative β-hCG	24,539	mIU/mL	Gestational-age dependent; nonpregnant <5
D-dimer	1.07	mg/L	≤0.50 (assay-dependent; nonpregnant)
Urinalysis – leukocyte esterase	Trace	—	Negative
Urinalysis – ketones	1+	—	Negative
Urinalysis – blood	2+	—	Negative
Urinalysis – WBC	6–10	/hpf	0–5
Urinalysis – RBC	4–5	/hpf	0–2
Urinalysis – casts	Moderate squamous and hyaline casts	—	None to few hyaline casts; no cellular casts
Urinalysis – bacteria	None	—	None
Urine culture	Negative	—	Negative / no growth
Blood culture	Negative	—	Negative / no growth
Parvovirus B19 PCR	Negative	—	Negative / not detected
Cytomegalovirus (CMV) PCR	Negative	—	Negative / not detected
Bartonella IgG/IgM	Negative	—	Negative
QuantiFERON-Gold	Negative	—	Negative
Monospot (mono screen)	Negative	—	Negative
Epstein-Barr virus (EBV) IgG	Positive (prior infection)	—	Negative (seronegative)
C-reactive protein (CRP)	1.2	mg/L	0-3
Erythrocyte sedimentation rate (ESR)	49	mm/h	0–20 (adult female; pregnancy-dependent)
Lactate dehydrogenase (LDH)	326	U/L	140–280
Peripheral flow cytometry	No genetic mutations identified	—	No abnormal findings
Purified protein derivative (PPD)	Positive	—	Negative; interpretation is risk-dependent

Imaging findings were consistent with her clinical presentation. Chest radiography demonstrated scattered bilateral nodular densities, raising concern for pulmonary involvement (Figure 1). Pelvic ultrasound confirmed a single intrauterine pregnancy at seven weeks and three days of gestation with a small subchorionic hemorrhage (Figure 2). Given the elevated D-dimer and pregnancy status, CT pulmonary angiography was performed; this ruled out pulmonary embolism but revealed scattered subcentimeter ground-glass opacities in the lungs (Figure 3). Bilateral lower extremity ultrasound excluded deep vein thrombosis.

**Figure 1:**
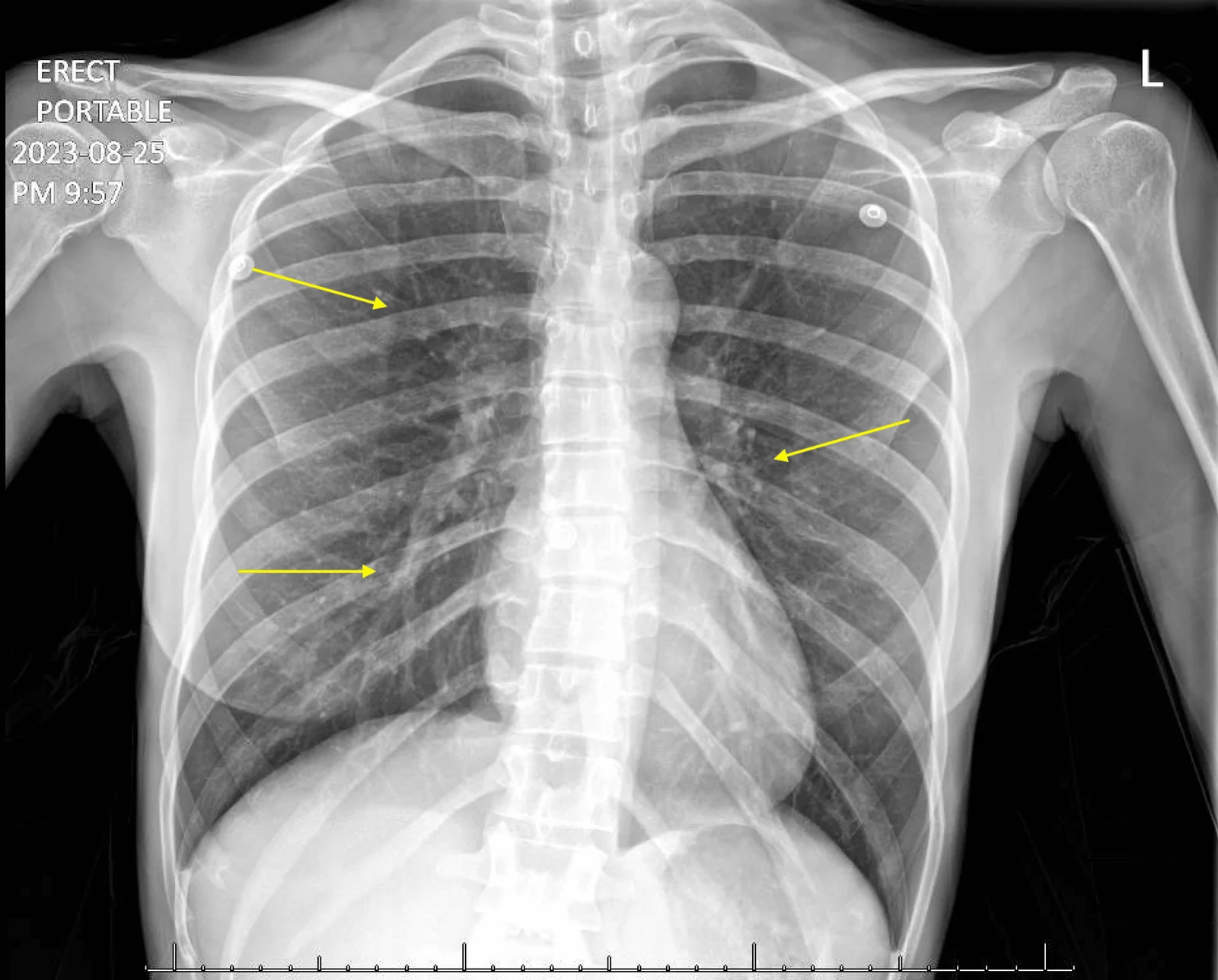
Initial Chest X-Ray Yellow arrows showing scattered nodular densities bilaterally, stable from prior.

**Figure 2:**
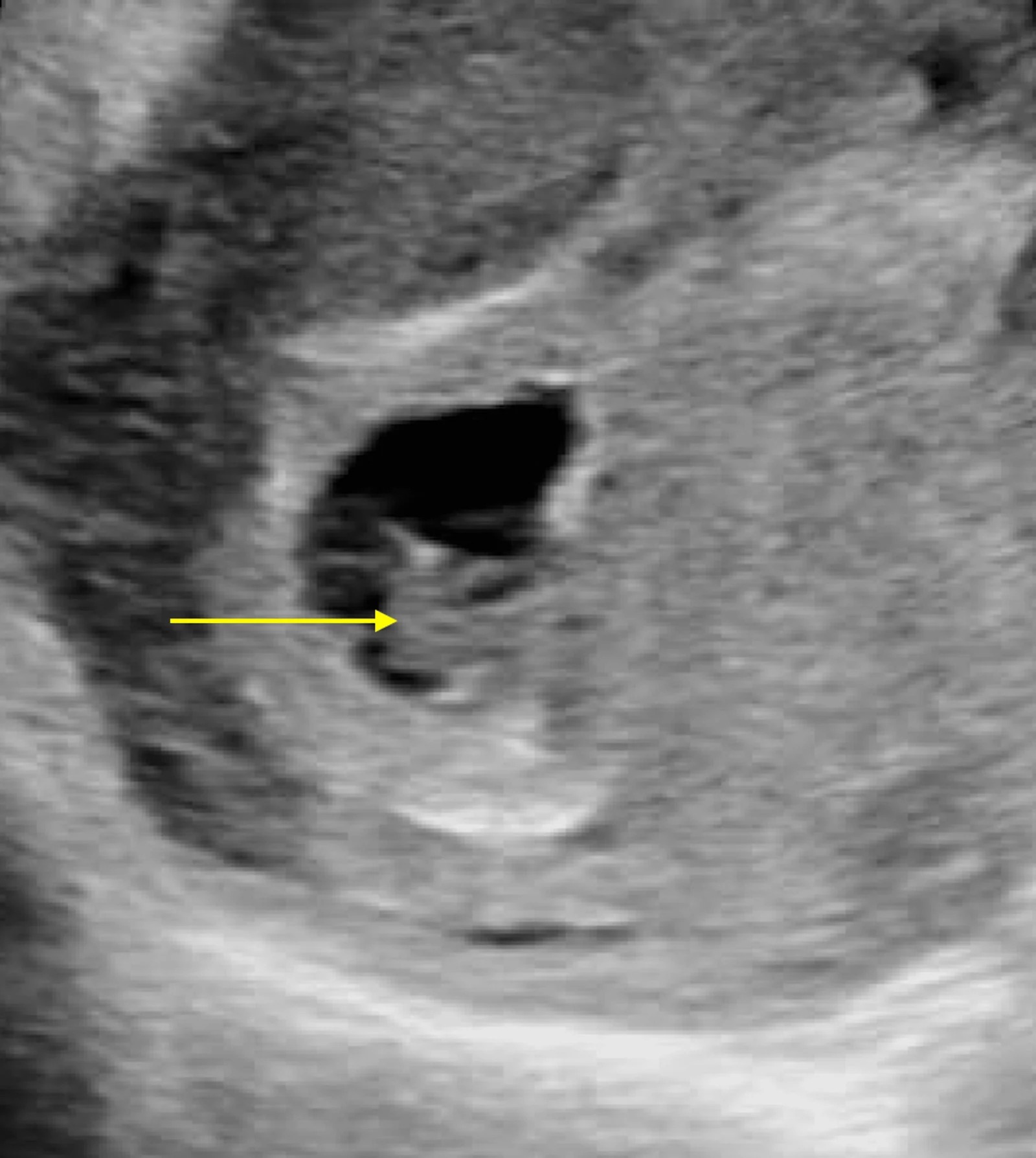
Pelvic Ultrasound Yellow arrow showing a single intrauterine pregnancy at seven weeks and three days of gestation with a small subchorionic hemorrhage.

**Figure 3:**
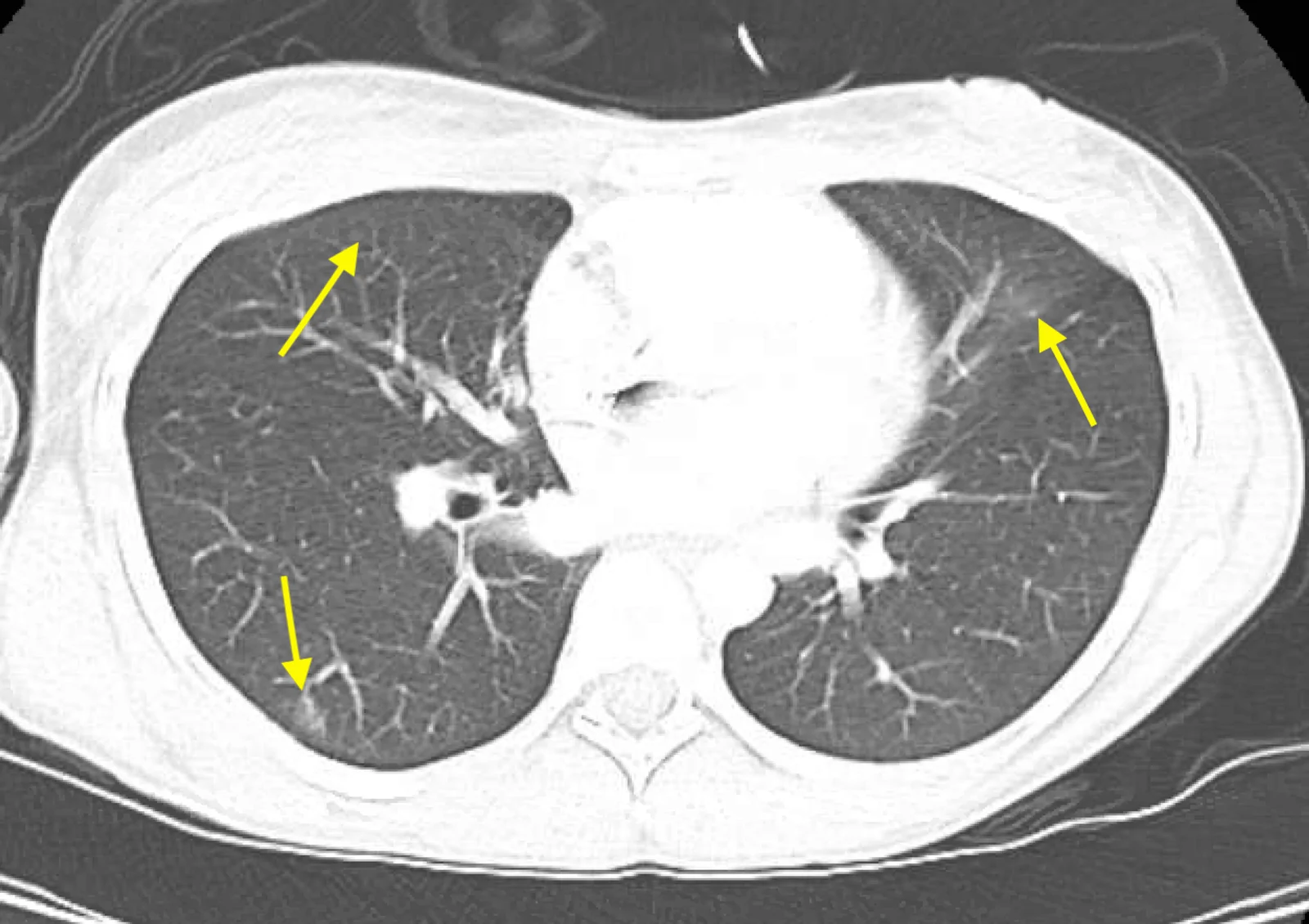
CT Chest with Pulmonary Embolism Protocol Yellow arrows showing scattered ground glass opacities

The patient was admitted, and consultations with infectious disease (ID) and obstetrics/gynecology (OB/GYN) were obtained. The ID team’s viral workup - including parvovirus B19 polymerase chain reaction (PCR), cytomegalovirus (CMV) PCR, Bartonella IgG/IgM, QuantiFERON-Gold, and a mono screen - was negative except for a positive Epstein-Barr virus (EBV) IgG, indicating prior infection. Inflammatory markers showed a normal C-reactive protein level but elevated erythrocyte sedimentation rate and lactate dehydrogenase levels. Hematology/oncology was consulted to evaluate malignant etiologies for the leukopenia and lymphadenopathy; peripheral flow cytometry revealed no genetic mutations, and biopsy of the posterior cervical lymph nodes was subsequently ordered. Over the course of a week-long hospitalization, her symptoms improved, with no further night sweats and diminishing neck pain. Following successful biopsy, she was discharged on prenatal vitamins and ferrous sulfate, with instructions to follow up with her primary care physician and oncology to review the biopsy results.

Approximately one week after discharge, pathology findings were consistent with Kikuchi-Fujimoto lymphadenopathy. Histologic findings revealed large patches of necrosis consisting of bright eosinophilic fibrinoid deposits, apoptotic cells, histiocytes, small lymphocytes, and plasma cells (Figures 4, 5). The histiocytes represented the majority of the infiltrating cells, and many showed crescentic nuclei. Neutrophils and eosinophils were absent, constituting a distinctive characteristic of this entity (Figure 6). Oncology recommended no further treatment or workup, advising reassessment only if symptoms recurred. The patient subsequently gave birth to her third child without complications or additional symptoms.

**Figure 4:**
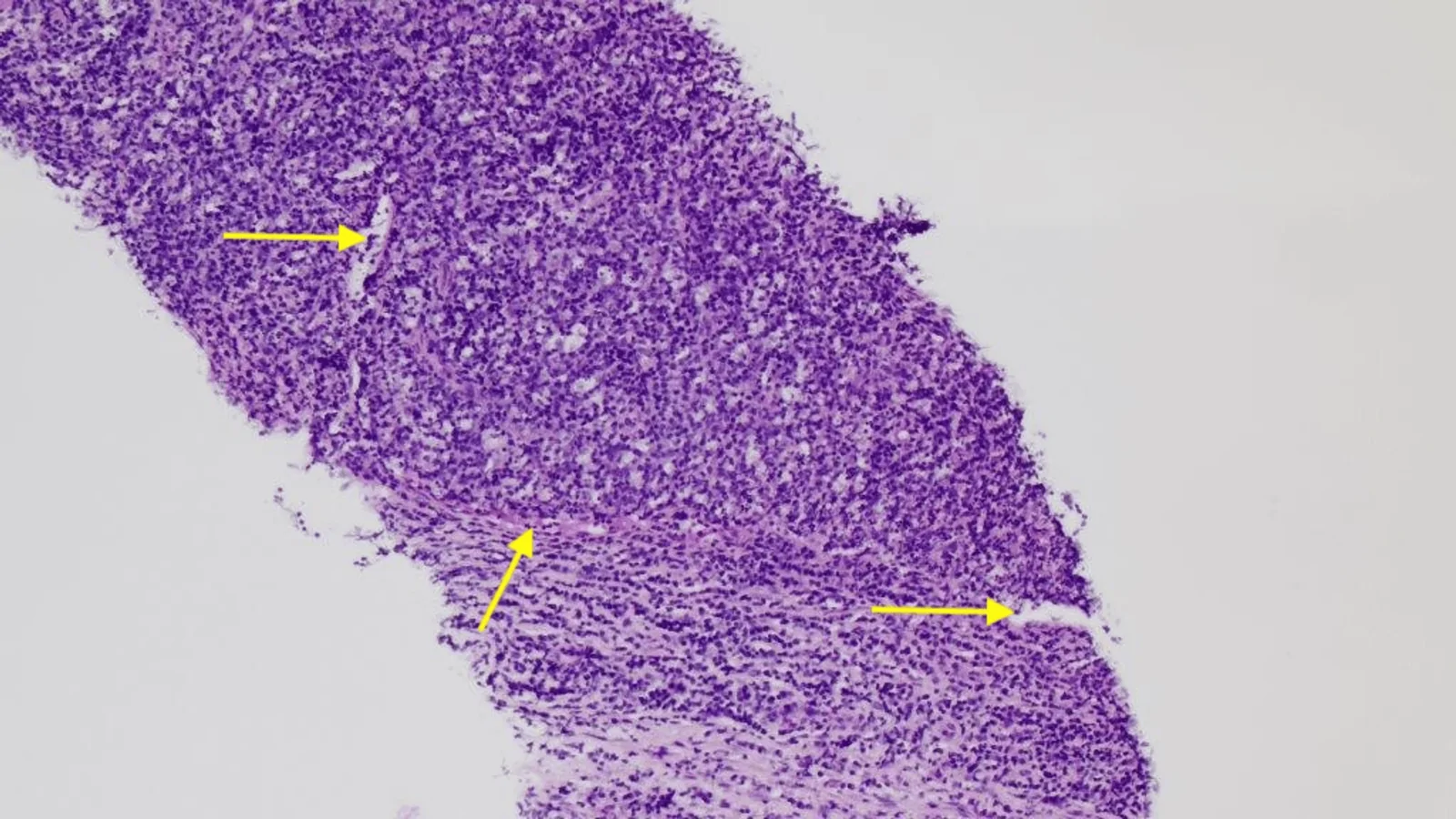
Supraclavicular Lymph Node Yellow arrows showing large patches of necrosis consisting of bright eosinophilic fibrinoid deposits, apoptotic cells, histiocytes, small lymphocytes, and plasma cells (H&E, ×40).

**Figure 5:**
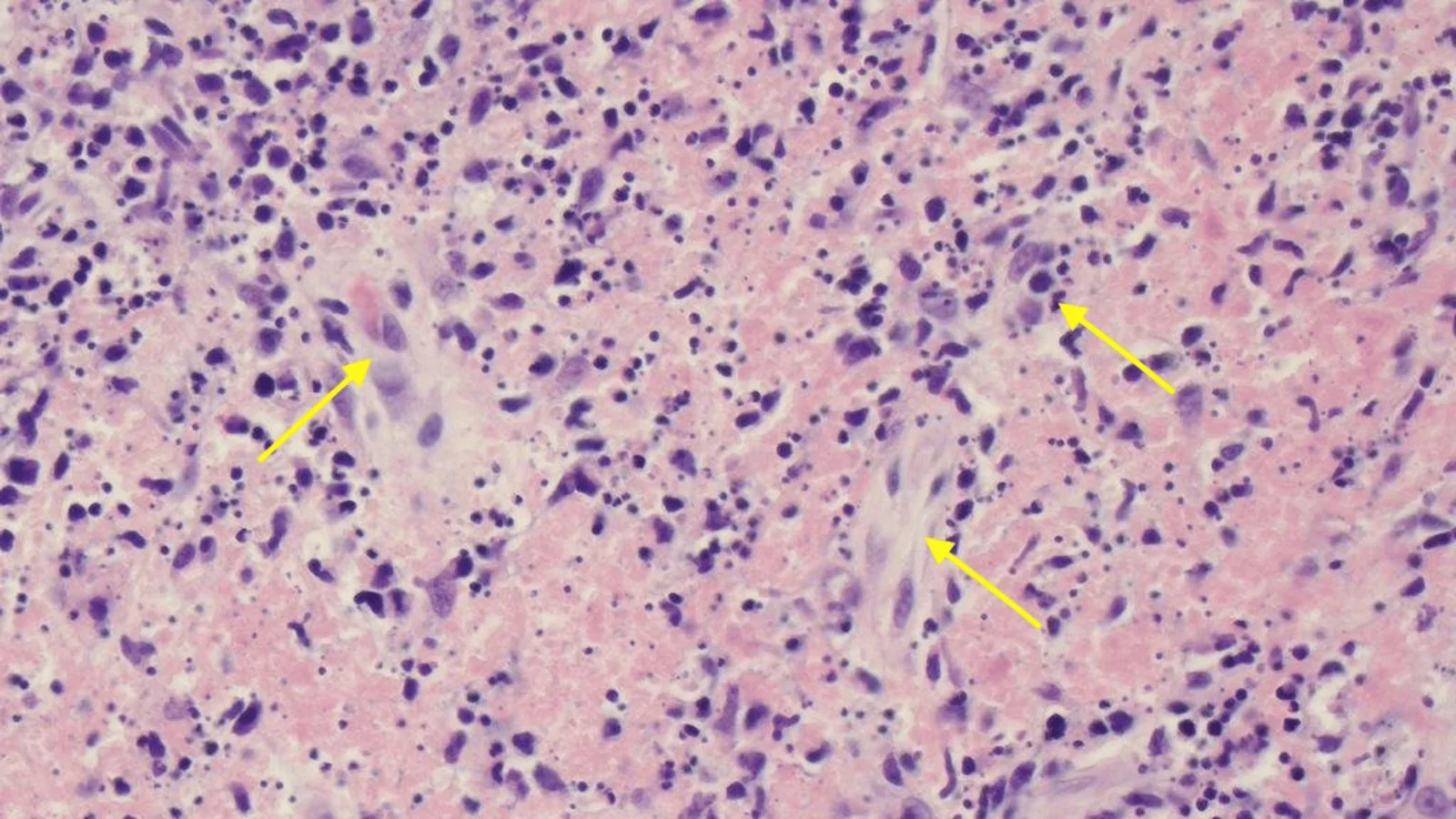
Supraclavicular Lymph Node 40x Yellow arrows showing eosinophilic fibrinoid deposits, apoptotic cells, histiocytes, small lymphocytes, and plasma cells (H&E, 40x).

**Figure 6:**
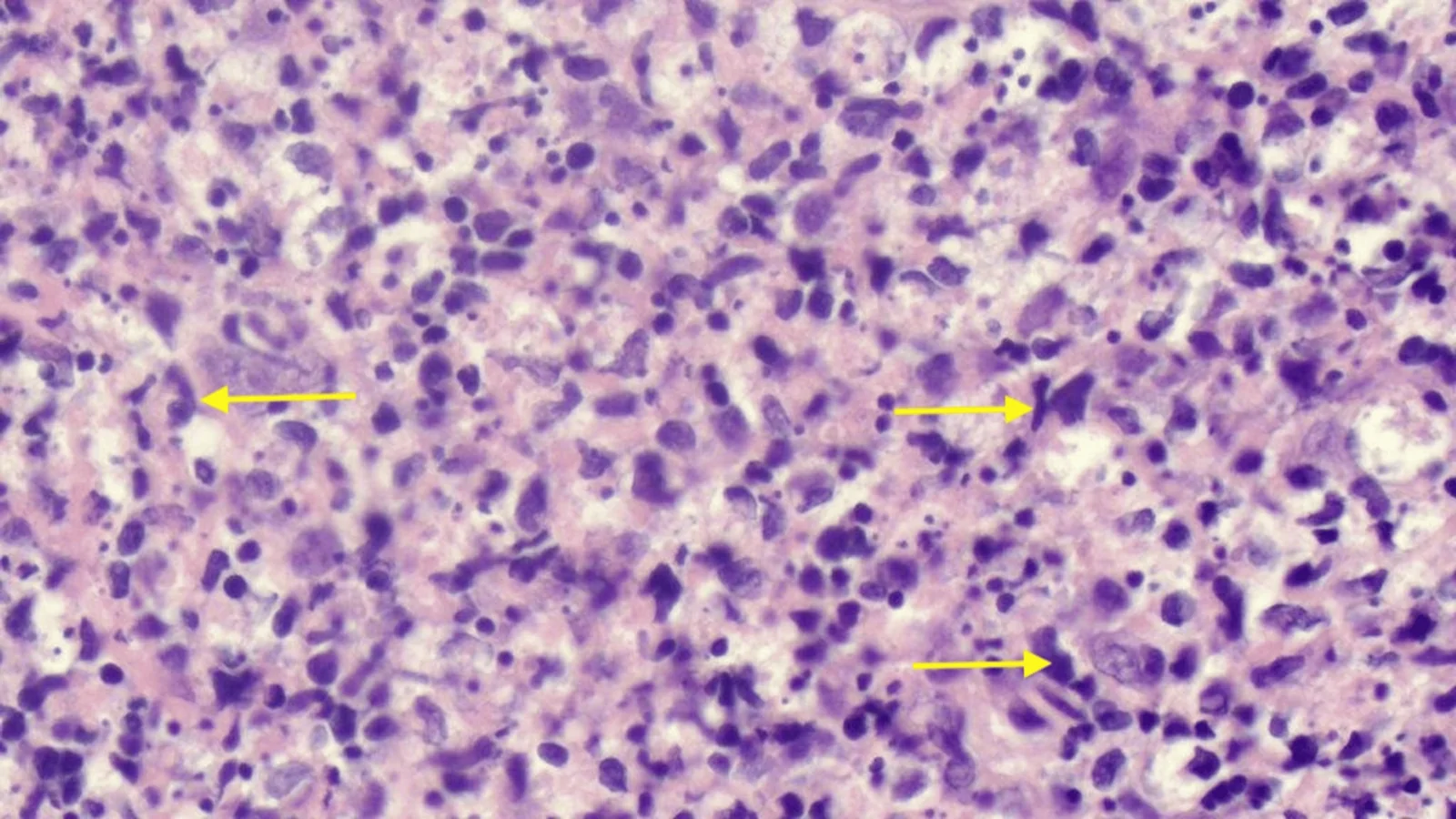
Aggregates of Histiocytes with Crecentic Nuclei and Apoptosis 40x Yellow arrows showing histiocytes with crescentic nuclei.  Neutrophils and eosinophils were absent, constituting a distinctive characteristic of this entity (H&E, 40x).

## Discussion

The diagnosis and management of KFD present a unique challenge, particularly in atypical cases where the clinical presentation overlaps with more severe conditions such as infection, autoimmune disease, and malignancy. Most patients with KFD present with unilateral, painful, mobile, nonsuppurative lymphadenopathy, but our patient exhibited diffuse, bilateral lymphadenopathy, a less common presentation seen in only 20% of cases [1]. Her diagnosis was further complicated by night sweats, fatigue, fever, and a false-positive PPD test, which broadened the differential diagnosis to include tuberculosis, other infections, and malignancy.

In pregnant patients such as ours, lymph node biopsy is considered a safe diagnostic step that poses no risk to the fetus, although high-dose nonsteroidal anti-inflammatory drugs (NSAIDs) should be avoided for post-procedure pain due to their association with premature closure of the ductus arteriosus.

KFD progresses through three distinct histopathologic stages: proliferative, necrotizing, and xanthomatous. The proliferative stage is characterized by the absence of neutrophils and eosinophils, along with follicular hyperplasia. The necrotizing stage involves karyorrhexis of histiocytes without destruction of the lymph node architecture. The xanthomatous stage marks the regression of necrosis and formation of foamy histiocytes. Immunohistochemistry further aids diagnosis, typically revealing histiocytes positive for myeloperoxidase and CD68, with sparse B cells and CD8-positive T cells [1]. Differentiating KFD from SLE-associated lymphadenitis is critical, as SLE requires prompt immunosuppressive treatment; classic findings in SLE lymphadenitis include DNA deposits in vessel walls, vasculitis, and hematoxylin bodies [1].

KFD generally resolves spontaneously within six months, with symptomatic management centered on over-the-counter anti-inflammatory medications. In severe cases, corticosteroids may be used, though their safety in pregnancy warrants consideration given a mild association with cleft lip and palate [2]. After resolution, patients should be closely monitored for the potential development of SLE.

KFD in pregnancy is rare, with only a handful of cases reported. In 2006, a 28-year-old woman presented at eight weeks’ gestation with a four-year history of cough and lymphadenopathy requiring intravenous steroids; her course was complicated by postpartum hemorrhage [3]. In 2008, a pregnant African American woman with biopsy-confirmed KFD experienced a miscarriage and later developed SLE [4]. A 2020 case from Cuba described a woman with KFD who had a prolonged 41-week gestation and an eutocic delivery [5]. In 2022, a pregnant woman initially misdiagnosed with EBV infection was subsequently found to have biopsy-confirmed KFD after steroid treatment failed [6]. Our patient’s presentation mirrored aspects of these cases, including pancytopenia, unsuccessful steroid treatment, and first-trimester onset. Unlike the previously reported cases, however, our patient’s infectious workup was positive for EBV IgG, suggesting a potential role for EBV in triggering KFD.

We reviewed the literature for a possible link between EBV and KFD. A 2001 study reported two pediatric cases of concurrent KFD and active EBV infection; histopathologic examination confirmed KFD with EBV DNA/membrane protein in both cases, and one child relapsed after initial improvement due to persistent EBV infection [7]. A 2010 report described a 16-year-old male who presented with fever and lymphadenopathy; biopsy confirmed KFD, and serologic testing showed active EBV infection, with an uncomplicated course that resolved without treatment [8]. A study from Taiwan identified EBV DNA in 10 KFD biopsy specimens by PCR, with variable gene expression levels suggesting a differential host response to the virus; the exact mechanism linking the two disease processes has not yet been established [9].

## Conclusions

Kikuchi-Fujimoto disease is a rare, self-limiting condition of unclear etiology that is typically benign, but its occurrence during pregnancy adds significant complexity to diagnosis and management. Our patient’s presentation - bilateral lymphadenopathy, night sweats, fatigue, fever, and a positive PPD test - illustrates the diagnostic challenges posed by KFD and the broad differential diagnosis it requires clinicians to consider. The positive EBV IgG further suggests a potential viral trigger, a connection noted in other cases but one that remains poorly understood.

This case underscores the importance of including KFD in the differential diagnosis of lymphadenopathy, particularly in high-risk populations, and the need for a comprehensive infectious and autoimmune workup to rule out serious conditions such as SLE or malignancy. Early recognition with appropriate lymph node biopsy is essential to avoid unnecessary treatment for more severe pathologies, and management during pregnancy requires careful attention to balancing symptomatic relief with fetal safety. Although KFD typically resolves spontaneously, ongoing monitoring remains essential given its potential association with autoimmune disease, including SLE. In our patient, a favorable outcome was achieved with no recurrence or complications following delivery, demonstrating that KFD in pregnancy can be successfully managed with vigilant follow-up and multidisciplinary care. Further research is needed to clarify the pathogenesis of KFD, particularly its relationship with viral infections such as EBV, in order to improve treatment strategies for this rare condition.
